# Inhibiting aberrant seizure-induced neurogenesis by temozolomide improves cognitive impairments associated with long-term amygdala kindling

**DOI:** 10.3389/fnins.2025.1626118

**Published:** 2025-09-03

**Authors:** Travis J. Francis, Brady S. Reive, Hugo Lehmann, Neil M. Fournier

**Affiliations:** Department of Psychology, Trent University, Peterborough, ON, Canada

**Keywords:** adult neurogenesis, pattern separation, memory, plasticity, kindling, seizure, epilepsy, hippocampus

## Abstract

In the adult dentate gyrus, new neurons are continuously generated and integrated into the existing circuitry where they play a crucial role in maintaining important functions related to learning and memory. Seizures not only robustly increase levels of hippocampal neurogenesis but can also induce aberrant migration and functional development of these new neurons, which has been hypothesized to promote network excitability and epileptogenesis. However, the contribution of new neurons to the development of epilepsy-related cognitive impairments remain unclear. Here, we investigated whether suppressing this abnormal elevation in neurogenesis that occur with seizures could prevent the emergence of cognitive dysfunction and behavioral deficits associated with chronic epilepsy. Using the long-term amygdala kindling model (consisting of 99 electrical stimulations), we demonstrate that initiating cyclic treatment with the DNA-alkylating agent temozolomide (TMZ) during a period of heightened neurogenic activity can reduce aberrant hippocampal neurogenesis and rescue impairments in a contextual fear discrimination task known to depend on functional neurogenesis. In addition, TMZ treatment also prevented object recognition memory deficits after kindling. Together, our findings suggest kindled seizures trigger the production of new neurons that can effectively rewire and interfere with hippocampal circuit function which can contribute to the development of chronic cognitive and behavioral deficits as seen in both patients with epilepsy and other rodent models. Thus, strategies that can selectively reduce aberrant adult neurogenesis may serve as a novel approach to treat cognitive deficits associated with epilepsy.

## 1 Introduction

Temporal lobe epilepsy is one of the most common focal seizure disorders, accounting for ~60% of all individuals with epilepsy (Wiebe, 2000). Apart from experiencing recurrent seizures, many patients are also at a higher risk for developing debilitating changes in cognitive functions, such as attention, language, and memory (Oyegbile et al., 2004; Helmstaedter and Witt, 2017; Novak et al., 2022). Among these cognitive issues, those involving learning and memory are the most frequent complaints of both children and adults with temporal lobe epilepsy and are a major source of reduced quality of life (Fisher et al., 2000; Elsharkawy et al., 2012). However, the neurobiological mechanisms that underlie the memory-associated impairments in chronic epilepsy are not well understood.

The dentate gyrus has long been implicated in the pathophysiology of temporal lobe epilepsy. The dentate gyrus is well positioned to exert a significant influence on information processing within the hippocampus. Both electrophysiological and imaging studies have shown dentate granule cells are considerably less excitable (Sloviter, 1991; Mody et al., 1992; Coulter and Carlson, 2007) and exhibit sparser activity than other hippocampal neurons (Chawla et al., 2005; Neunuebel and Knierim, 2012; Diamantaki et al., 2016). These unique characteristics enable these cells to play an important role in activities such as pattern separation (McHugh et al., 2007), a process that helps to enhance the precision of memory recall by reducing overgeneralization and interference that arises from the encoding and/or retrieval of similar experiences or memories (Kheirbek et al., 2012). The low excitability of the dentate gyrus can also further help to “gate” incoming inputs into the hippocampus and prevent pathological overexcitation from occurring (Lothman et al., 1992; Krook-Magnuson et al., 2015; Scharfman, 2019).

The dentate gyrus also stands out as one of the few adult brain regions involved in the ongoing production of new neurons in rodents and humans (for review, see (Moreno-Jimenez et al., 2021; Kempermann et al., 2023). As adult-born dentate granule cells mature and integrate into the surrounding neural circuitry, they exhibit a period of heightened excitability and plasticity (Schmidt-Hieber et al., 2004; Ge et al., 2007; Li et al., 2017; Huckleberry and Shansky, 2021) which enables them to support a variety of hippocampal functions. Multiple studies have demonstrated that seizure activity can dramatically but transiently increase levels of hippocampal neurogenesis (Parent et al., 1998; Scott et al., 1998; Nakagawa et al., 2000; Jessberger et al., 2007; Parent, 2007; Ammothumkandy et al., 2022), with a subset of new neurons exhibiting aberrant development, such as the maintained presence of basal dendrites, axonal sprouting, and ectopic migration into the hilus (Walter et al., 2007; Pierce et al., 2011; Murphy et al., 2012; Botterill et al., 2015; Singh et al., 2015; Chen et al., 2022). Furthermore, many of these new neurons appear to be more excitable and receive fewer inhibitory inputs than their mature counterparts, suggesting that they may participate abnormally within hippocampal circuits (Althaus et al., 2019; Lybrand et al., 2021). Although acute seizures or injuries are associated with an initial increases in proliferation and neurogenesis, levels of neurogenesis tend to decline during chronic epilepsy (Hattiangady et al., 2004; Jessberger and Parent, 2015; Nasluzova et al., 2020). This biphasic effect of seizures on adult neurogenesis points to pervasive and ongoing alterations within the neurogenic niche during chronic epilepsy.

While much of this research has focused on the role of seizure-induced hippocampal neurogenesis in epileptogenesis, there is growing evidence that the abnormal integration of adult-born neurons can have broader consequences on cognitive function. Kindling refers to the process whereby daily electrical stimulation of forebrain structures, such as the amygdala, results in a gradual intensification of electrographic and behavioral seizure activity (Goddard et al., 1969). Once an animal has reached the criterion of being “fully kindled” (i.e., it has displayed three or more consecutive Class 5 or higher generalized convulsive seizures), it will continue to respond to each stimulation with a generalized seizure even after a stimulation free period lasting several months (Goddard et al., 1969). Because of these features, the kindling model has been widely used to investigate the pathophysiology of human temporal lobe epilepsy and explore potential cellular and network changes that underlie the emergence of interictal behavioral dysfunction (see Kalynchuk and Fournier, 2009). Several studies have shown that electrical kindling causes a significant increase in hippocampal neurogenesis particularly during the early stages of seizure development (Parent et al., 1998; Scott et al., 1998; Nakagawa et al., 2000; Smith et al., 2006; Fournier et al., 2010; Jafari et al., 2012; Fournier et al., 2013). In addition, kindling can also impair a variety of hippocampal-mediated behaviors in rodents, including spatial learning, fear conditioning, and anxiety (Adamec, 1990; Hannesson et al., 2001; Fournier et al., 2009; Botterill et al., 2014; Fournier et al., 2020). Our own investigation has found that these behavioral deficits are greatest in the period that follows the enhanced proliferation and network integration of adult-born neurons during kindling, suggesting that these early seizure-generated neurons might function abnormally within the existing hippocampal circuitry and contribute to circuit dysfunction (Fournier et al., 2013). However, direct evidence linking aberrant seizure-evoked neurogenesis to the emergence of cognitive deficits associated with chronic epilepsy is lacking.

To date, most studies exploring the effects of kindling on neurogenesis have employed short-term kindling procedures, in which the process of kindling terminates shortly after the animals have met the criterion of being “kindled.” This typically occurs after 15 to 30 stimulations to amygdaloid nuclei, such as the basolateral amygdala (Racine, 1972b; Sutula and Kotloski, 2017). However, when kindling continues beyond this point, motor convulsive seizures increase in severity and interictal epileptiform spikes become more pronounced. We and others have shown that the associated behavioral effects of kindling are more robust and reliable when the number of stimulations exceeds those used in short-term kindling procedures—particularly after animals have received between 70 and 100 stimulations (Kalynchuk et al., 1997; Botterill et al., 2015; Fournier et al., 2020; Zahra et al., 2022; Song et al., 2024). Thus, the extended or long-term kindling model more accurately captures the progressive nature of epilepsy, including the emergence of interictal and cognitive impairments as seen in clinical populations. Given this, we set out to address whether suppressing seizure-induced hippocampal neurogenesis might mitigate the development of cognitive impairments that occurs with electrical long-term kindling.

In the present study, we addressed this question by administering the DNA alkylating agent Temozolomide (TMZ), which has been shown to suppress levels of adult hippocampal neurogenesis (Garthe et al., 2009; Niibori et al., 2012) and improve cognitive function through inhibition of aberrant neurogenesis following experimentally-induced stroke in mice (Cuartero et al., 2019). To determine the functional impact of TMZ treatment, rats were tested on a behavioral pattern separation task, a procedure that is well known to require intact neurogenesis and recruitment of adult-born neurons. Our findings show that TMZ is effective in suppressing neurogenesis, and importantly, prevents the development of memory deficits in kindled animals. These results highlight the importance of targeting aberrant neurogenesis as a novel strategy to mitigate cognitive impairments associated with epilepsy.

## 2 Materials and methods

Male Long-Evans (Charles Rivers, Montreal, QB, Canada) rats weighing between 200 and 250 g at the time of arrival were used as the subjects. Prior to surgery, rats were group housed in conventional rectangular polypropylene cages with standard laboratory bedding and kept on a 12:12 h light:dark cycle with light on at 0700 h local time. Ambient temperature was held at 20°C. Food and water were available ad libitum throughout the experiment. All procedures were approved by Trent University's Animal Care Committee (Animal Use Protocol: 28020) and in compliance with guidelines set out by the Canadian Council on Animal Care. Efforts were made to minimize the number of animals used in the experiment.

### 2.1 Stereotaxic surgery

A total of 70 rats underwent stereotaxic surgery. Rats were individually anesthetized with isoflurane (5% initial, 2–2.5% maintenance) and treated an analgesic (carprofen, 10 mg/kg, i.p.) to minimize pain and discomfort during post-surgery recovery. The rat was then secured in a stereotaxic apparatus and an incision was made along the midline of the scalp and the overlying skin retracted. A single bipolar electrode (MS 303/2-B, Plastics One, Roanoke VA) was implanted into the left basolateral amygdala at the following coordinates: −2.8 mm posterior to bregma, +5.00 mm medial/lateral, and −8.5 mm ventral to surface of the skull (Paxinos and Watson, 1998). The electrode assembly was secured with stainless steel screws and dental acrylic. The incision site was cleaned with 10% povidone iodine solution and a topical antibiotic cream (Polysporin^®^) was applied to reduce risk of infection. Rats were placed in heated recovery cage until they were fully recovered and then returned to their home cage. Twenty-four hours later, rats received daily treatment with carprofen (10 mg/kg, i.p.) daily for the first 3 to 5 days after surgery to reduce signs of pain and inflammation. Rats were allowed at least 1 week of recovery from surgery before start of kindling.

### 2.2 Kindling procedure

Rats were randomly assigned to one of three conditions: long-term kindling, long-term kindling with TMZ treatment, or sham controls. In this context, long-term kindling refers to the cumulative or total number of electrical stimulations, which generally exceeds 60 stimulations (Kalynchuk and Fournier, 2009). Unlike conventional kindling procedures, the number of stimulations during long-term or extended kindling exceeds the number required for animals to reach a fully kindled state, which is defined as a minimum of three consecutive Class 5 or higher generalized convulsive seizures. As such, long-term kindling is considered to model more closely the progressive epileptogenic changes and associated comorbidities that are seen in chronic epilepsy (Kalynchuk and Fournier, 2009).

The kindling procedure consisted of 75 or 105 electrical stimulations delivered by an isolated pulse stimulator (Model 2100, A-M Systems, Sequim WA USA) to the left basolateral amygdala (1 msec biphasic square wave pulses, 60 p.p.s for 1 s). The amplitude of the current was set to 800 μA (peak-to-peak). The intensity of this electrical stimulation has been shown to be well above the threshold to evoke an epileptiform afterdischarge in the amygdala (Racine, 1972a). Electrical stimulations were delivered three times daily with a minimum of 3 h between consecutive stimulations. Stimulations were delivered for 4 consecutive days followed by 3 days of no-stimulations to accommodate the treatment cycles of TMZ (see below). Sham controls underwent the same handling procedure as the kindled groups except no current was delivered.

The behavioral convulsion induced after each stimulation was recorded using a modified Racine scale (Racine, 1972b): Class 0 – freezing; Class 1 – orofacial automatisms; Class 2 – orofacial automatisms with head nodding; Class 3 –unilateral forelimb clonus; Class 4 – rearing with bilateral forelimb clonus; Class 5 – rearing with bilateral forelimb clonus followed by falling; Class 6 – multiple Class 5′s. Rats were returned to their home cage once all convulsive behaviors subsided. Rats were considered to be “kindled” after achieving three consecutive Class 5 or higher convulsions.

### 2.3 Temozolomide treatment

Temozolomide (TMZ) has been widely used as a pharmacological approach to investigate the effects of reduced adult neurogenesis on behavior in mice and rats. TMZ (MedChem Express, CAS: 825622-93) is a DNA alkylating prodrug with high blood-brain barrier permeability that is commonly used as an oral treatment for high-grade glioblastoma multiforme. TMZ (2.5 mg/ml) was freshly prepared each day by dissolving in ice-cold sterile 0.9% (w/v) saline (pH 5.5–6.0). The TMZ solution was then vortexed (~30 s) and briefly sonicated (50 W, 5 s) in an ice bath. The TMZ solution was sterile filtered before use to remove remaining insoluble residues and remained on ice during injections.

**Figure 1:**
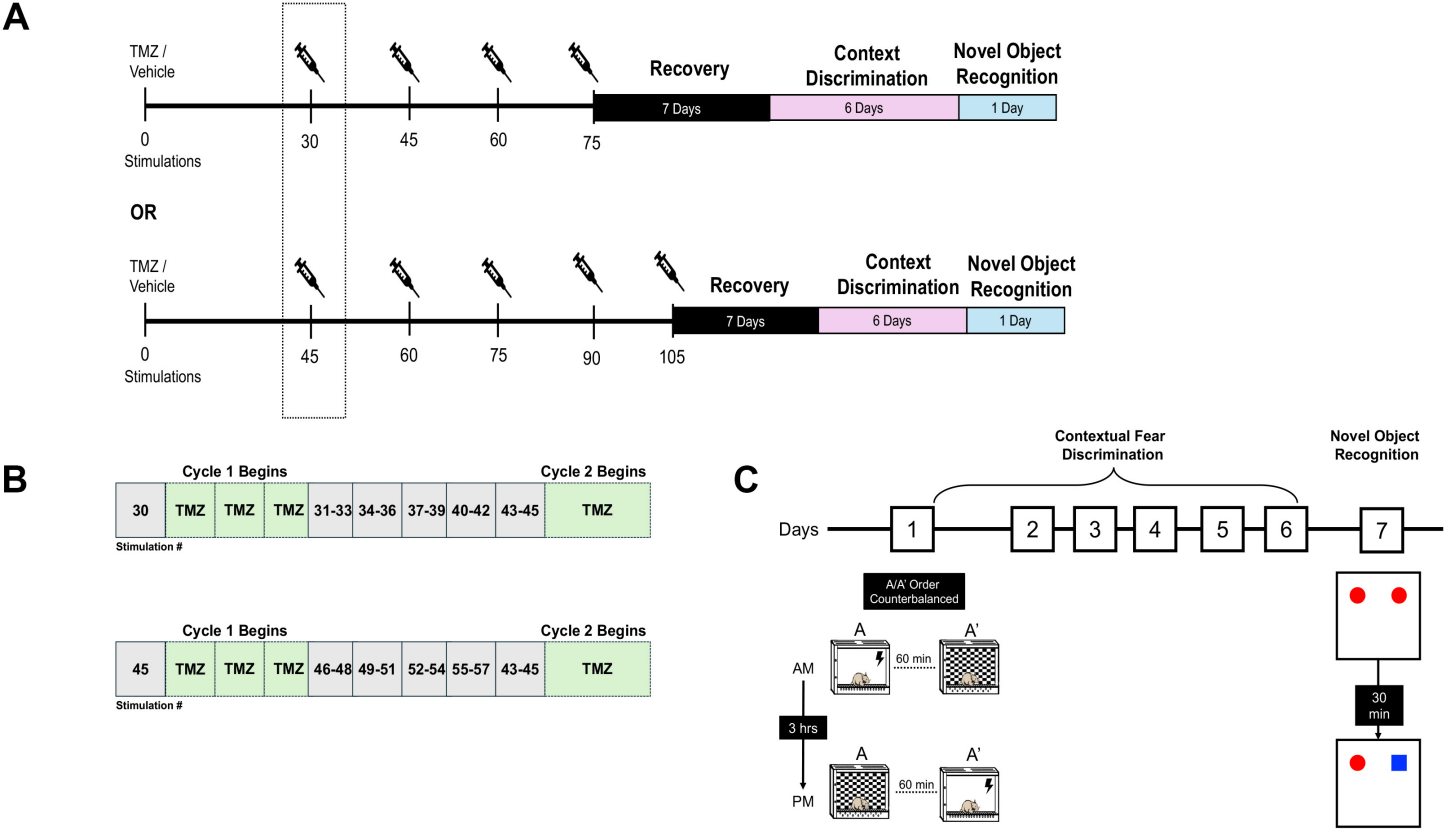
Experimental timeline and behavioral testing. **(A)** Rats underwent amygdala kindling to a total of 75 or 105 stimulations. TMZ (25 mg/kg, i.p) or vehicle (0.9% saline) was administered twice daily for three consecutive days beginning after the 30th or 45th stimulation. Kindling resumed 24 h after each treatment cycle (e.g., between cycle 1 and 2, etc.). **(B)** Schematic depicting the alternating cycles of TMZ treatment and stimulation blocks used in the experiment. **(C)** Diagram illustrating the procedures involved in contextual discrimination learning and the novel object recognition task. Behavioral testing began 1 week after the last TMZ treatment cycle (see Methods for additional details).

In this study, rats were administered twice-daily injections of TMZ (25 mg/kg, i.p.) or saline, with injections spaced at least 4 h apart, for three consecutive days with a 4-day kindling period immediately following the last TMZ treatment. The first cycle of TMZ treatment for the kindled groups began after the 30th or 45th electrical stimulation to target the period of enhanced neurogenesis during kindling. Subsequent treatment cycles continued until the animal received a total of either 75 or 105 stimulations (Figures 1A, B). This ensured that both TMZ and saline-treated kindled groups achieved a similar number of elicited motor convulsions during the kindling procedure. During the treatment free period, kindled and sham stimulations (3 stimulations per day) resumed over the next 5 days until the following cycle of TMZ or saline began. Kindled rats received 4 or 5 treatment cycles of TMZ or saline during kindling period. To minimize potential confounding issues related to TMZ treatment and/or indirect (postictal) effects from kindling on behavior, a 1-week recovery or rest period was given. Using this protocol, TMZ has been demonstrated to reliably inhibit adult neurogenesis without causing general impairments in the health of the animal (Garthe et al., 2009; Nokia et al., 2012; Bambico et al., 2015; Perez-Domper et al., 2017; Nickell et al., 2020).

### 2.4 Contextual fear discrimination

#### 2.4.1 Apparatus

Beginning 7 days after the final TMZ treatment, rats were exposed daily to two operant chambers that were identical in shape and size (25.4 cm x 25.4 cm x 36.5 cm) enclosed in a sound attenuating box. The chambers were equipped with a ventilation fan (set to 50%) to mask extraneous noise, and the internal light level was set at 100 lux. The chambers were modified to produce two similar contexts (Context A and Context A') that shared many common features, including Plexiglas sidewalls, a transparent front door and roof, an exposed stainless-steel grid floor. However, Context A' was further modified in that chamber walls were covered with a checkered plastic insert (made from ethylene vinyl acetate). Additionally, Context A was cleaned using Oxivir Five 16 Concentrate (1:16 dilution), whereas Context A' was cleaned each with 70% (v/v) ethanol that was scented with several drops of concentrated lemon extract. Foot-shocks were delivered through the grid floor, which was connected to a shock generator and scrambler (Ugo Basile, Varese, Italy). All training sessions were video recorded using a webcam placed above the conditioning chamber that was connected a computer installed with Any-Maze software (Stoelting Co., Wood Dale, IL USA).

#### 2.4.2 Training

Each rat was randomly assigned to receive shocks in either context A or A', with the opposite context (A' or A) being assigned as the no-shock (“safe”) condition. Context fear discrimination training was carried out over 6 consecutive days with rats receiving two exposures to each context (A and A') daily. For “shock” trials, the rat was placed into their assigned context (A or A') and following a 120 s acclimation period a single footshock (2 s, 1.0 mA) was delivered. The rat was removed 60 s later and returned to their home cage. For the “safe” trial, rats were placed into their designed context (A' or A) for 180 s and then returned to their home cage after completion of the trial. No shocks were given during the “safe” trials. There were four trials per day with two trials (shock or safe) in the morning (spaced 1–1.5 h apart) and another two trials (shock or safe) in the afternoon (spaced 1–1.5 h apart). The interval between the morning and afternoon sessions were ~3 to 4 h. The order of presentation of the shock and safe trials for the morning and afternoon sessions was randomized each day using a Latin-square design.

The percentage of time each rat spent freezing, defined as an absence of movement except those necessary for respiration, during the first 120 s of each trial was measured using an automated detection software (Any Maze) based on the following parameters: minimum freezing duration – 250 ms; freezing onset/offset threshold – 70 and 80 (no units), respectively.

#### 2.4.3 Object recognition test

Novel object recognition testing was carried using the same apparatus and procedures as described (Horsey et al., 2019). Briefly, the test consisted of two phases: a sample trial and a retention (test) phase. For the sample phase, each rat was allowed 5 min to freely an open field area that contained two identical objects. A retention test was conducted 30 min later. During this test, one of the objects previously presented was replaced by a new object. The rats were returned to the open field arena and allowed to explore for 5 min during the test phase. Object investigation was defined as sniffing (within 3 cm) or touching the object with the nose and/or forepaws. The time spent by the rats investigating each object, familiar or novel, during the test phase was recorded by a video camera and was scored by a researcher blind to the housing condition of each subject. A discrimination index (DI) was calculated as follows: DI = (novel object)/(novel object + familiar object) multiplied by 100, this ratio represents the time spent investigating the novel object expressed as a proportion of the total time spent investigating both objects. The open field arena was cleaned with 70% alcohol and air-dried prior to the next trial.

#### 2.4.4 Tissue preparation and immunohistochemistry

Rats were deeply anesthetized with sodium pentobarbital (240 mg/ml) and transcardially perfused with 0.9% physiological saline followed by ice-cold 4% (w/v) depolymerized formaldehyde that was freshly prepared. Brains were extracted and post-fixed in the same fixative for 48 h at 4°C prior to being sectioned at 50 μm on a Leica VT-1000 vibratome. All sections were stored at 4°C in 0.01% (w/v) sodium azide in a 0.1M phosphate buffered saline solution (PBS, pH = 7.5) until processed for immunohistochemistry.

Doublecortin (DCX) immunohistochemistry was carried on free-floating sections with all rinses and incubations carried out under gentle agitation as previously described (Horsey et al., 2019). Briefly, sections were incubated overnight in a primary antibody solution containing rabbit anti-DCX antibody (1:2000, Cell Signaling, #4064S), 5% (v/v) normal goat sera, 1% (w/v) bovine serum albumin, and 0.3% (v/v) Triton X-100 diluted in PBS at 4°C. The following day, the tissue sections were washed several times PBS and incubated with a biotinylated goat anti-rabbit IgG secondary antibody (1:500, MJX Biolynx) diluted in 0.3% Triton X-100 in PBS for 2 h at room temperature. The sections were then placed in avidin-biotin-peroxidase complex (1:200, Vectastain Elite ABC, MJX Biolynx) solution and immunolabeling was visualized using 0.02% (w/v) DAB, 2.5% (w/v) nickel ammonium sulfate, 0.083% (v/v) H_2_O_2_ in 0.175 M sodium acetate (pH = 7.0) to yield a bluish black product. After 20 min, the reaction was stopped by washing the sections several times in 0.1 M PBS. The sections were mounted onto glass slides (Superfrost Plus, Fisher Scientific) and left to air dry overnight. Slides were dehydrated through a series of alcohols, cleared in xylene, and coverslipped with DPX mounting medium. A separate well of brain sections was also stained with 0.1% cresyl violet, and electrode placements were verified for each animal using Paxinos and Watson stereotaxic atlas (1998). Animals with incorrectly positioned electrodes were removed from the study.

#### 2.4.5 DCX quantification

All quantifications were performed by an experimenter who was blind to the treatment condition of the subjects.

For each rat, 8 to 12 coronal sections were analyzed and the total number of DCX+ immature neurons was estimated using the optical fractionator method (West et al., 1991). Every sixth section was examined at 100X (oil immersion) magnification on a Nikon Eclipse 80i microscope equipped with a motorized stage and a computerized stereology system (Stereologer). DCX+ cells found within the dentate granule cell layer and the subgranular zone was counted and the total number of DCX+ cells was estimated using the following formula: *N*_Total_ = ∑Q = 1/ssf X *A*(*x,y* step)/*a*(*frame*) X *t*/*h*; where ∑Q is the number of cells counted; *ssf* is the section sampling fraction (1/6); *A*(*x,y* step) is the area associated with each *x,y* movement (200 μm); *a*(*frame*) is the area of the counting frame (5,250 μm^2^); *t* is the weighted average section thickness; and *h* is the height of the dissector (12 μm). A guard zone of 2 μm was used to avoid sectioning artifacts. The coefficient of error was calculated and all values < 0.15 were accepted. Due to the small number of hilar ectopic cells, stereological procedures were not performed and instead the total number of ectopic DCX+ cells was manually counted in a demarcated area containing the hilus.

To examine DCX labeling in the dorsal and ventral dentate gyrus, the mean area fraction of DCX-labeling within the dentate subgranular zone, granule cell layer, and inner molecular layer was quantified as a single region of interest (ROI). The subgranular zone was defined as a 50 μm thick band that extended from the hilar surface of the granule cell layer and captured the majority of DCX+ cells. The inner molecular layer was considered the first inner third of the molecular layer immediately above the dentate granule cell layer. Sections were imaged on a Nikon TiE inverted research microscope with NIS Elements software. Images were acquired and processed using ImageJ. The ROI was outlined at 10X magnification and threshold was set so that DCX+ cells could be easily delineated from background. The area of DCX immunoreactivity within the ROI was calculated and expressed as a percent total area of the dentate gyrus. DCX immunoreactivity was measured from 3 sections for the dorsal dentate gyrus (AP −2.92 to −3.60 mm) and 3 sections for the ventral dentate gyrus (AP −4.92 to −5.64 mm). The area fractions were then averaged across the dorsal and ventral ROIs to provide a single mean area fraction value.

#### 2.4.6 Statistical analyses

All statistical analyses were performed using SPSS (Statistical package for the Social Sciences v 26) and a *P* < 0.05 was considered significant for all tests. For contextual fear discrimination learning, a three-way repeated measures ANOVA was used to analyze group differences (sham vs. kindled vs. kindled/TMZ) on freezing levels with trial (shock vs. safe) and session (days 1 through 6) as the within subject factors. Paired *t-*tests and Sidak's *post hoc* tests, with corrections applied for multiple comparisons, were used to assess group differences where appropriate. For analysis of DCX immunolabeling, a one-way ANOVA was used to examine for group differences in the number of DCX+ cells in the dorsal and ventral dentate gyrus.

## 3 Results

Only rats with electrodes that correctly targeted the left basolateral amygdala and who showed typical progression of kindling-induced epileptogenesis and behavioral convulsions proceeded to the behavioral testing portion of this study. During the study, 1 rat died due to complications from surgery and 8 rats were removed due to improper electrode placement or failure to develop motor convulsions (Class 5 or higher) during kindling. In addition, one rat treated with TMZ exhibited weight loss (>30%) during the study, which resulted in their removal. As a result, the final groups were kindled (*n* = 21), kindled plus TMZ (*n* = 19), and sham (non-kindled) controls (*n* = 20), respectively (Supplementary Figure 1).

Kindling produced a typical progression of limbic motor convulsions in all rats. There was no difference in the mean number of stimulations required to evoke the various classes of behavioral seizures (Class 1 through 6) for kindled rats with treated TMZ and those treated with saline (data not shown). Furthermore, the mean number of stimulations required to elicit the first Class 5 seizure was 15.7 (SEM ± 4.8) for the kindled group and 14.6 (SEM ± 6.4) for the kindled group receiving TMZ was also not statistically significant. In line with previous studies, no spontaneous seizures were observed in any rats throughout the duration of the experiment (Pinel and Rovner, 1978; Fournier et al., 2013, 2020).

Because group differences in seizure frequency and/or severity could account for subsequent behavioral effects we aimed at exploring, we attempted to limit this possibility by ensuring comparable rates of kindling between TMZ and saline-treated kindled rats. This was accomplished by adjusting the kindling procedure resulting in subset of rats from each respective group receiving either 75 or 105 electrical stimulations, which ensured that the number of convulsive seizures and hence seizure burden would be similar across groups. The total number of Class 5 or higher seizures was found to 69.9 (SEM: 6.90, range 39–87) for the kindled group and 71.6 (SEM: 3.05, range: 61–87) for the kindled group that received TMZ. There were also no significant within group differences observed for rats that received 75 or 105 stimulations on any of the histological or behavioral measures examined in this study (data not shown). Therefore, for each treatment condition (saline vs. TMZ), kindled rats that received 75 or 105 stimulations were pooled together for all subsequent analyses.

### 3.1 Kindling-induced neurogenesis is blocked by temozolomide

**Figure 2:**
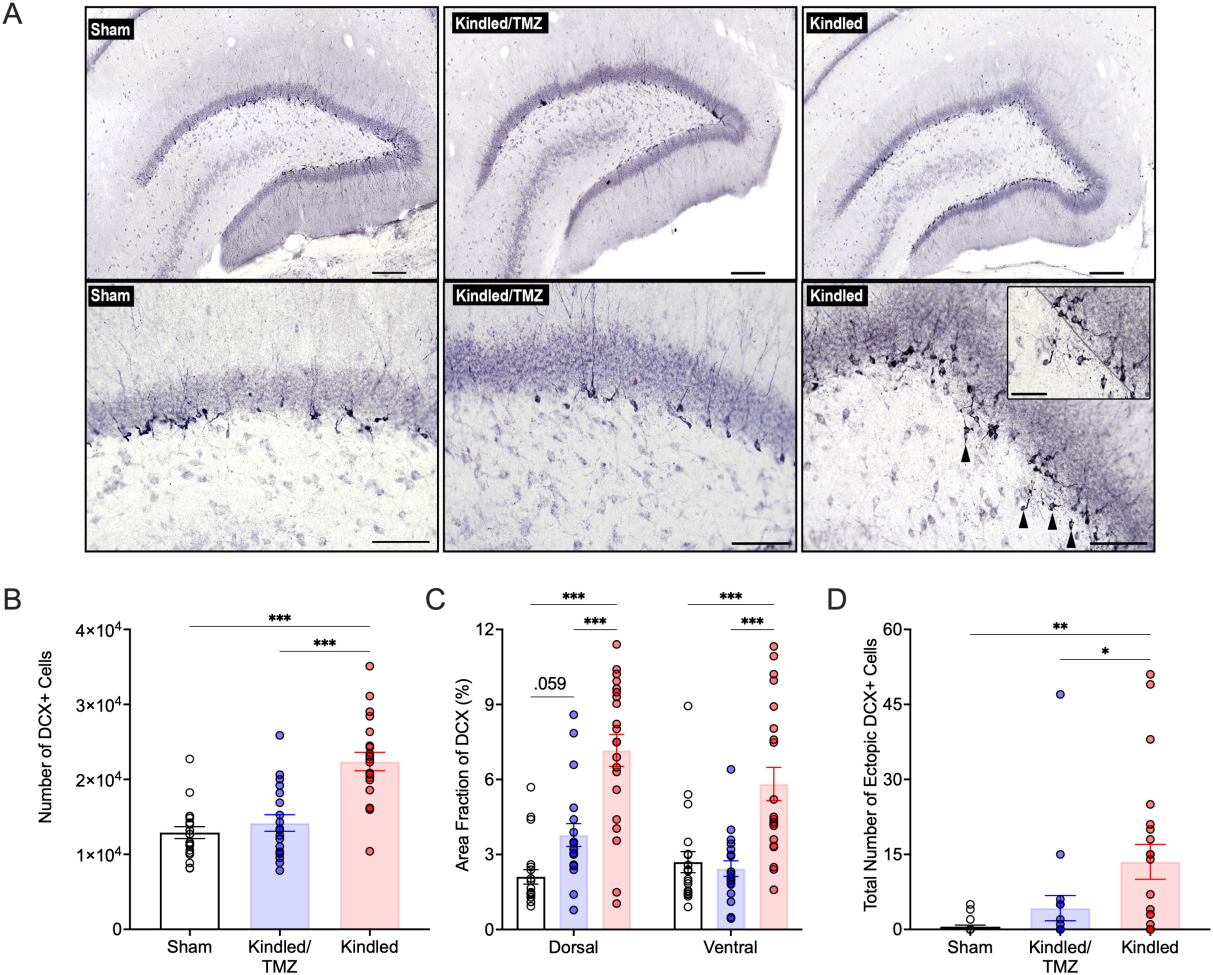
Effect of TMZ on aberrant kindling-induced hippocampal neurogenesis. **(A)** Representative images of doublecortin (DCX) immunolabelling in the adult dentate gyrus from non-kindled (sham) controls, kindled rats that received TMZ, and kindled rats that received saline. Ectopic DCX+ cells in the hilus are indicated by black arrowheads. Upper Panels: scale bar, 200 μm; Bottom Panels: Scale bar, 100 μm. Inset: higher magnification image showing ectopic DCX+ cells in the hilus. The border of the dentate granule cell layer and subgranular zone is marked with a black dotted line. Scale bar, 50 μm. **(B)** Unbiased stereological estimates of the number of DCX+ cells in the dentate granule cell layer and subgranular zone. Kindled rats treated with saline had significantly more DCX+ cells than both sham controls and kindled rats treated with TMZ. ***P < 0.001 (significantly different from sham controls and TMZ treated kindled rats). **(C)** Area fraction of analysis of DCX immunolabeling in the dorsal and ventral hippocampus. Saline-treated kindled rats displayed a greater area fraction of DCX+ labeling compared to sham controls and TMZ treated kindled rats in both the dorsal and ventral hippocampus. ***P < 0.001 (significantly different from sham controls and TMZ-treated kindled rats). **(D)** Total number of ectopic DCX+ cells in the hilus, summed across all analyzed brain sections per rat. Saline-treated kindled rats had significantly higher numbers of hilar DCX+ cells than sham controls. TMZ treatment significantly reduced the number of hilar DCX+ cells following kindling. **P < 0.01, and *P < 0.05 (significantly different from sham controls and TMZ treated kindled rats). Data are presented as mean ± standard error of the mean.

To assess for changes in levels of adult hippocampal neurogenesis after kindling, we examined the immature neuronal marker doublecortin (DCX). DCX is a microtubule-associated protein associated with neuronal differentiation and neurite elongation, which is exclusively expressed in immature dentate granule cells from 1 day to about 4 weeks post-mitosis (Brown et al., 2003; Plumpe et al., 2006). As expected, DCX-immunolabeled cells were found predominately clustered along the border of the dentate granule cell layer and subgranular zone (Figure 2A). Using unbiased stereological cell counting procedures, we found a significant effect of group on the total number of DCX+ cells in the dentate gyrus [ANOVA, *F*(2, 57) = 23.98, *P* < 0.001, η^2^ = 0.46]. The results presented in Figure 2 show that kindled rats had an almost 1.7-fold increase in the number of DCX+ compared to sham controls (Sidak, *P* < 0.001). As shown in Figure 2B, multicycles of TMZ treatment markedly suppressed the effect of kindling on hippocampal neurogenesis as the number of DCX+ cells in this group was significantly lower than rats that received kindling only (Sidak, *P* < 0.002) and approached the levels of sham controls (Sidak, *P* = 0.788).

Given that the dorsal (septal) and ventral (temporal) hippocampal regions are anatomically and functionally distinct, we also examined if kindling might differentially affect neurogenesis across the septotemporal axis of the dentate gyrus. Compared to sham controls, the mean area fraction of DCX immunoreactivity was increased by 3.3-fold in the dorsal dentate gyrus (Figure 2C) and ~2-fold in the ventral dentate gyrus (Figure 2C) after kindling [Dorsal: two-way ANOVA, *F*(2, 57) = 28.3, *P* < 0.001, η^2^ = 0.49, ω^2^ = 0.47; Ventral: two-way ANOVA, *F*(2, 57) = 14.45, *P* < 0.0001, η^2^ = 0.33, ω^2^ = 0.31]. However, the kindling-induced increase in DCX immunoreactivity was significantly attenuated by TMZ treatment. Although TMZ reduced overall decrease in DCX levels across the dorsal and ventral dentate gyrus after kindling, this reduction was more pronounced in the ventral dentate gyrus (kindled/TMZ: dorsal vs. ventral, paired *t*-tests, *P* = 0.009), where the mean area fraction of DCX labeling was no longer significantly different from that of sham controls. In contrast, DCX immunoreactivity in the dorsal dentate gyrus remained slightly elevated for the TMZ group compared to sham controls, though this difference only approached statistical significance [*P* = 0.059]. Taken together, our findings suggest that cyclic treatment of TMZ (beginning after the 30th or 45th stimulation) can effectively suppress kindling-induced hippocampal neurogenesis.

### 3.2 Temozolomide prevents aberrant hippocampal neurogenesis during kindling

Seizures not only enhance the generation of new neurons but can also promote aberrant morphological development and migration of these cells. DCX+ cells in the non-kindled controls had typical granular morphology and generally displayed only a single apical dendritic process that reached the molecular layer before ramifying into additional smaller branches. However, the morphology of DCX+ cells from kindled rats displayed more irregular-shapes, characterized by flattened and elliptical-like cell bodies, and showed more expansive dendritic growth and branching within molecular layer. Remarkably, many of these morphological changes were reduced in kindled rats that received TMZ. While there was also some DCX+ cells that ectopically migrated into the hilus after kindling [ANOVA, *F*(2, 57) = 7.13, *P* < 002, η^2^ = 0.20, ω^2^ = 0.17, Figure 2D], this was almost completely suppressed after TMZ treatment. Together these results suggest that kindling promotes an increase in aberrant neurogenesis that can be prevented by multicycle treatment with TMZ.

### 3.3 Temozolomide reverses kindling-induced contextual discrimination deficits

To investigate if blocking the increase in aberrant hippocampal neurogenesis that occurred with kindling could prevent learning deficits, animals underwent training in a contextual pattern separation task (see Figure 1C for task procedure). This task required the animal to discriminate between two highly similar conditioning contexts: a shock-paired context (A) and a slightly modified context that was never paired with a foot shock (A'). We performed a three-way mixed design ANOVA with Day (1 through 6) and Context (A vs. A') as the within subject factors and Group (sham vs. kindled vs. kindled/TMZ) as the between subject factor to examine for changes in freezing across the conditioning sessions. The results revealed a significant Group by Context interaction [RM-ANOVA, *F*(2, 50) = 8.02, *P* < 0.001, partial η^2^ = 0.24] and a marginally significant three-way interaction involving Group, Day, and Context [RM-ANOVA, *F*(10, 250) = 1.89, *P* = 0.077, partial η^2^ = 0.07]. Significant main effects for Context [*F*(1, 57) = 9.49, *P* < 0.003, partial η^2^ = 0.16], Day [*F*(5, 286) = 37.74, *P* < 0.001, partial η^2^ = 0.43], and Group [*F*(2, 50) = 7.67, *P* < 0.001, partial η^2^ = 0.23] were also found.

**Figure 3:**
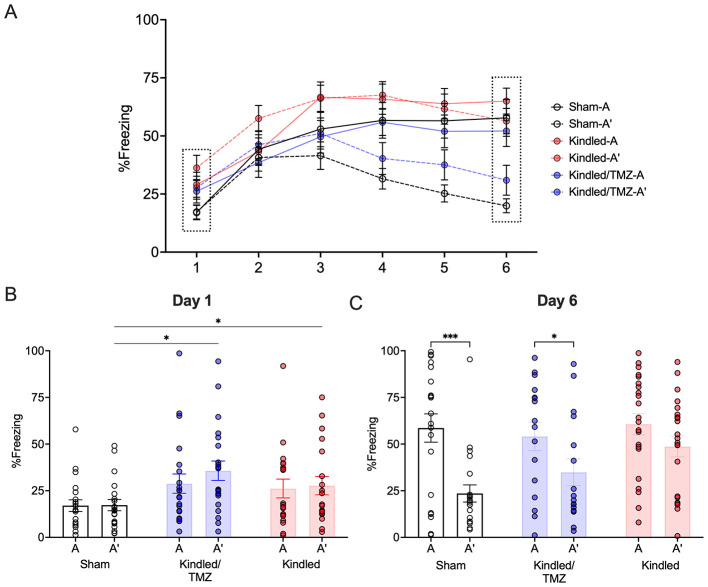
Effect of TMZ treatment during kindling on contextual discrimination learning. Rats were trained for 6 days to distinguish between an aversive and safe context by associating context A with a footshock and context A' with no footshock. **(A)** The mean percentage of freezing to the shock-paired context (context A) and safe context (context A') across 6 days of conditioning. **(B, C)** The mean percentage of freezing to the shock-paired and safe contexts on the first and last day of conditioning. On Day 1, kindled rats, regardless of treatment, exhibited higher levels of freezing to both contexts. *P < 0.05 (significantly different from sham controls). By Day 6, sham controls and TMZ-treated kindled rats froze significantly less to the shock-paired context **(A)** than the safe context (A') indicating successful discrimination. In contrast, kindled rats treated saline continued to display similar levels of freezing to both contexts. ***P < 0.001, *P < 0.05 (significantly different from context A). Data are presented as mean ± standard error of the mean.

For context A (the shock only context), all groups showed equivocal levels of freezing to this context over the 6 days of conditioning (RM-ANOVA, All *Fs* < 1.88, All *Ps* > 0.162). In contrast, analysis of context A' (the no shock context) found group differences in freezing across the conditioning sessions [RM-ANOVA, Group by Day, *F*(10, 285) = 2.87, *P* < 0.002, partial η^2^ = 0.10, Figure 3A]. As shown in Figure 3B, kindled animals irrespective of treatment displayed initially higher freezing scores to context A' than the sham controls on the first day of conditioning (Sidak, *P* < 0.015). However, by the following day, all groups showed similar freezing to context A'. Over the remaining conditioning days, kindled rats continued to show higher levels of freezing to context A' (the no shock) compared to sham controls, however, this difference was only significant on Days 5 and 6 of training when contrasted with kindled rats that received TMZ (Sidak All Ps < .026). Importantly, there was no difference in freezing to the no shock context (A') for sham controls and kindled rats that received TMZ over the remainder of these conditioning sessions suggesting that both groups demonstrated similar levels of freezing to this context.

To further examine if kindled rats had difficulty distinguishing between contexts A and A' throughout all conditioning sessions, we conducted a series of paired *t*-tests to determine when significant differences in freezing to the two contexts emerged. Sham controls showed a significant decrease in freezing to context A' compared to context A on day 3. This difference persisted and remained statistically significant until day 6 (paired *t*-tests, All *Ps* < 0.019, All Cohen's *ds* = 0.497 to 1.17, Day 6 - Figure 3C). In contrast, kindled rats that received saline displayed high levels of freezing to both context A and A' across all training session indicting an overall difficulty in discriminating between the two contexts. Interestingly, treatment with TMZ appeared to mitigate the effect of kindling. Similar to sham controls, kindled rats that received TMZ showed reduced freezing to the no-shock context A' when contrasted with the shock-paired context A with this difference beginning one day after sham controls (Day 4, paired t-test, P < .038, Cohen's d = 0.51) and remaining until the end of conditioning (paired *t*-tests, All *Ps* < 0.013, Cohen's *ds* = 0.55 to 0.74, **Day 6 –** Figure 3C). These finding suggest that kindled rats may have difficulties discriminating between the two contexts leading to increased fear generalization. However, chemical ablation of neurogenesis during kindling appears to alleviate this deficit resulting in improved contextual discrimination learning.

**Figure 4:**
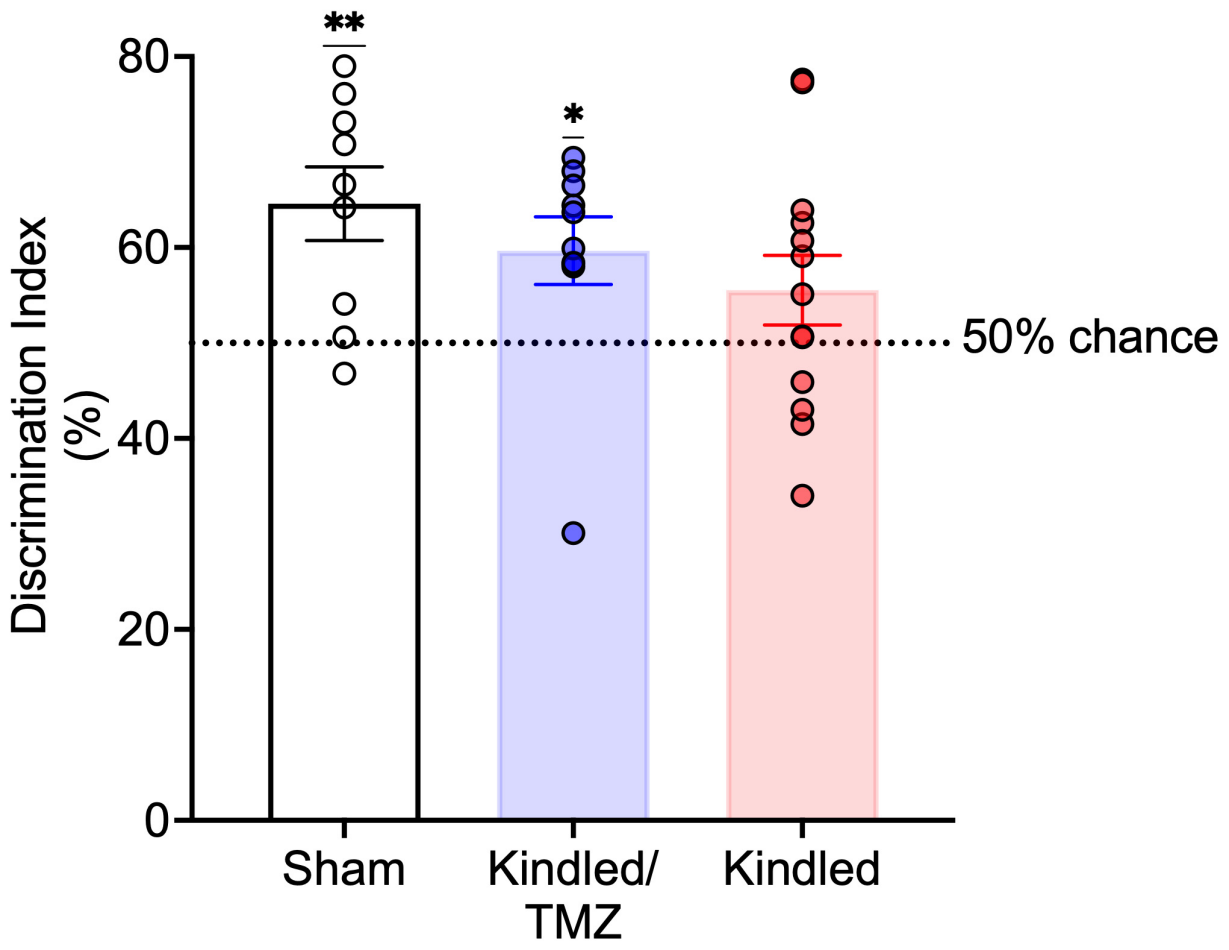
Effect of TMZ treatment during kindling on novel object recognition recall. Discrimination index examined during a 3 min recall test. TMZ-treated kindled rats and sham controls spent significantly more time exploring the novel object than familiar object at levels that were greater than chance. In contrast, saline-treated kindled rats did not show a preference for the novel object that was greater than chance. The broken line represents chance performance (50%). Data are presented as mean ± standard error of the mean. **P < 0.004, *P < 0.05 (significantly different from chance performance).

To examine if blocking seizure-induced neurogenesis might impact performance on another behavioral task, a subset of animals underwent testing for novel object recognition (sham *n* = 11; Kindled *n* = 10; Kindled/TMZ *n* = 10). Following a 30-minute retention interval, a one-sample t-test was used to examine whether the discrimination index significantly differed from 50% (chance level). As shown in Figure 4, both sham controls and the kindled rats received TMZ appeared to successfully discriminate the novel object from the familiar object during the recall test [one-sample *t*-tests, sham, *t*(8) = 3.77, *P* < 0.005, Cohen's *d* = 1.26; kindled/TMZ, *t*(9) = 2.73, *P* < 0.023, Cohen's *d* = 0.86]. However, the discrimination index for kindled group that received saline did not differ significantly from chance [one-sample *t*-test, *t*(9) = 1.51, *P* = 0.155, Cohen's *d* = 0.42].

## 4 Discussion

In the present study, we examined the role that seizure-induced hippocampal neurogenesis plays in the development of cognitive impairments associated with chronic epilepsy. To achieve this, we electrically evoked seizures from the basolateral amygdala and administered repeated cycles of the DNA alkylating agent TMZ to chemically suppress reactive neurogenesis during kindling. While TMZ has been typically given as a chemotherapeutic treatment for glioblastoma, it has been recently used as a pharmacological strategy to alter levels of neurogenesis in adult rodents (Garthe et al., 2009; Nokia et al., 2012; Egeland et al., 2017; Nickell et al., 2020; Zong et al., 2022; Yu et al., 2023).

Our data show that repeated treatment cycles with TMZ can potently reduce the rise in hippocampal neurogenesis that accompanies electrical kindling. TMZ treatment also appeared to prevent seizure-induced anomalies in neurogenesis. Our work further revealed that suppressing kindling-evoked neurogenesis can ameliorate deficits in contextual discrimination learning and object recognition memory to performance levels that were comparable to non-kindled controls. Taken together, our findings add to growing evidence pointing to a key contribution of aberrant hippocampal neurogenesis in underlying aspects of cognitive impairments associated with chronic epilepsy.

### 4.1 TMZ blocks aberrant neurogenesis during kindling

We confirm past work (Parent et al., 1998; Scott et al., 1998; Fournier et al., 2010, 2013; Chen et al., 2023) that electrical kindling of the amygdala dramatically increases the number of immature neurons (DCX+ cells) generated in the dentate gyrus. Although birth-dating experiments with the thymidine analog BrdU was not performed, we have previously shown that the increased proliferation or birth of new neurons tends to be highest during the early stages of electrical kindling—beginning round 30th stimulation and peaking at the 45th stimulation—when rats begin to regularly display overt motor convulsions in response to stimulation and meet the criteria for being classified as “kindled” (Fournier et al., 2010, 2013). During this early stage of kindling, heightened network stimulation appears to drive neural stem cell activity leading to a rapid expansion of proliferating pools of neuroprogenitor cells. However, as kindling stimulations extend beyond this point, repeated evocation of seizures lead to changes in the morphology and connectivity of adult-born neurons, along with a decline in proliferation and neurogenesis toward baseline values. For example, in both this study and our previous work (Fournier et al., 2010, 2013) we found that several DCX+ cells exhibit migration deficits with displacement into the hilus. In addition, several DCX+ cells located in in the granule cell layer and subgranular zone displayed aberrant structural alterations, including formation of hilar basal dendrites and increased dendritic branching. These findings are reminiscent of what has been commonly reported in multiple rodent chemoconvulsant models of status epilepticus, such as pilocarpine and kainic acid (Scharfman et al., 2000; Hattiangady et al., 2004; Jessberger et al., 2007; Walter et al., 2007; Pierce et al., 2011; Hester and Danzer, 2013; Jain et al., 2019) suggesting that seizure stimulation—despite being evoked through different experimental methods—appear to produce similar disruptions in proliferation, maturation, and connectivity of newborn neurons.

Several lines of evidence show that adult-born neurons (between 2 to 6 weeks of age postmitosis) exhibit greater intrinsic excitability and plasticity than their mature counterparts (Ash et al., 2023). As a result, immature neurons at this stage of development may be especially vulnerable to the effects of pathological stimulation, such as seizure activity. In support of this, Lybrand et al. (2021) and others (Singh et al., 2015; Hosford et al., 2016; Althaus et al., 2019; Zhou et al., 2019; Chen et al., 2023) found that when seizure activity occurred throughout this critical window it drove ectopic migration, aberrant morphological development and accelerated integration of new neurons. This window overlaps with the period of extended kindling in our study and aligns with our finding that repeated seizures evoked during this time can disrupt the normal development of new neurons.

We hypothesized that TMZ treatment during this critical period might be an effective strategy to reduce aberrant neurogenesis and improve hippocampal function. Our findings support this. First, we found that TMZ significantly blunted kindling-evoked neurogenesis, with the number of DCX+ cells being comparable to sham controls at the end of kindling. Second, TMZ also prevented the generation of aberrant newly generated neurons as there was an almost complete suppression of ectopically displaced DCX+ cells into the hilus along with a reduction in DCX+ cells that exhibited abnormal morphology. These findings suggest that most aberrant new neurons born during kindling appear to be generated during this early wave of enhanced neural stem cell/progenitor activity.

Although TMZ was effective in arresting kindling-induced neurogenesis across the septotemporal axis of the hippocampus, this effect appeared greater in the ventral hippocampus compared to the dorsal hippocampus. The exact reason for this difference is unclear. However, Egeland et al. (2017) reported in mice that 6-week of TMZ treatment caused a greater reduction in neurogenesis in the ventral hippocampus compared to the dorsal hippocampus suggesting that the ventral hippocampus may be more vulnerable to the effects of antimitogenic agents such as TMZ. Since the ventral hippocampus displays overall lower rates of neurogenesis and proliferative activity than the dorsal hippocampus (Wu et al., 2015), it is possible that these differences could enable the impact of TMZ on neurogenesis to be more apparent. In fact, we found that while TMZ reduced overall levels of neurogenesis in the dorsal and ventral hippocampus after kindling, DCX+ immunolabelling still trended toward being significantly elevated within the dorsal hippocampus compared to sham controls. Regardless, our findings confirm the anti-mitogenic and suppressive actions of TMZ on hippocampal neurogenesis as previously reported in multiple rodent studies further highlighting its usefulness as a tool to study neurogenesis in the adult brain (Garthe et al., 2009; Egeland et al., 2017; Che et al., 2021).

### 4.2 Role of aberrant hippocampal neurogenesis in cognitive impairment

The accurate migration, development, and integration of new neurons is crucial for hippocampal function. Thus, a central question of this study was to investigate whether the ongoing production of abnormally integrated new neurons during kindling could explain, at least in part, the development of cognitive and behavioral deficits that are seen in chronic epilepsy. To address this, we used a contextual fear discrimination task which requires the animal to distinguish between a context associated with a mild footshock and a similar context that was never paired with the shock. Contextual discrimination has been shown to depend on pattern separation and correlates with levels of intact adult hippocampal neurogenesis (Sahay et al., 2011; Niibori et al., 2012; Tronel et al., 2012; Winocur et al., 2012; Denny et al., 2014; Miller and Sahay, 2019).

Our findings clearly show that repeated seizures induced by electrical kindling caused pronounced deficits in contextual discrimination learning. For example, while non-kindled controls could effectively discriminate between the shock and non-shock contexts by Day 3 of training, kindled rats were unable to do so and displayed high levels of freezing to both contexts across the training sessions. Enhanced fear generalization has been linked to behavioral pattern separation deficits and suggests repeated kindled seizures may sensitize neural circuits involved in threat detection and defensive responses. This parallels clinical observation of elevated interictal anxiety and fear among patients with mesial temporal lobe epilepsy (Hermann et al., 1982; Heilman, 2021; Rauh et al., 2022). In fact, we have previously shown that long-term amygdala kindling can also produce exaggerated anxiety and enhanced fear generalization in rodents (Kalynchuk and Fournier, 2009; Fournier et al., 2020).

Most importantly, we found that impairments in context discrimination were ameliorated by TMZ treatment. Kindled rats that received TMZ showed discriminative freezing to both contexts by Day 4 of training, indicating that inhibition of seizure-induced neurogenesis appears to mitigate discriminative fear deficits produced by kindling. In this context, our finding that TMZ appeared to produce greater suppression of kindling-evoked neurogenesis within the ventral hippocampus might be particularly relevant. The ventral hippocampus is known to be highly implicated in emotional regulation and in the disambiguation between threatening and safe contexts (Fournier and Duman, 2013; Kheirbek et al., 2013; Besnard et al., 2020). It is possible that aberrant neurogenesis within the ventral hippocampus could disrupt the balance between excitatory and inhibitory circuits which could lead to overgeneralized fear responses (Li et al., 2024). While speculative, our findings suggest that by reducing seizure-induced neurogenesis, particularly within the ventral hippocampus, TMZ may help restore circuit stability which could improve pattern separation and reduce inappropriate fear generalization across contexts (Besnard and Sahay, 2016). In addition to this, we also observed a protective effect of TMZ on another behavioral task, the novel object recognition test. We found that kindled rats treated with TMZ demonstrated comparable levels of recall as sham controls during testing, whereas non-treated kindled rats showed impairments in recognition memory. Object recognition is typically associated with extra-hippocampal areas, such as the perirhinal cortex. This suggests that TMZ preservation of object recognition memory may extend beyond its effect on suppressing seizure-induced neurogenesis. However, object-based and novelty-related information is still relayed to the hippocampus to support overall object processing (Kinnavane et al., 2016). Therefore, by preventing seizure-induced neurogenesis within the hippocampus, TMZ may help offset the formation of aberrant network changes that disrupt interactions between the hippocampus and other brain regions, such as the perirhinal cortex, during object learning. Taken together, our findings highlight an important role of seizure-induced neurogenesis as a potential mechanism underlying the development of epilepsy-associated cognitive impairments, and that targeting this process through chemical ablation with TMZ can prevent cognitive decline associated with chronic seizures.

## 5 Limitations

Our findings suggest that chemical ablation of hippocampal neurogenesis can prevent the development of seizure-induced hippocampal network changes that underlie cognitive deficits seen with kindling. However, it is possible some of the behavioral effects observed could be mediated by TMZ's effect on other neurogenic regions, such as the amygdala, olfactory bulbs, hypothalamus, and striatum. Prior research has shown that prolonged seizures can increase proliferation and migration of new neurons into the olfactory bulbs and other forebrain regions (Parent et al., 2002). Our own unpublished findings have also found that amygdala kindling induces DCX expression in regions such as the piriform cortex. Whether this reflects active proliferation and differentiation of new neurons is not known. Nonetheless, the effects of seizure on other neurogenic regions and their potential contribution to epilepsy-related cognitive and behavioral impairment is an important area of research that warrants further attention. In addition, the possibility of off-target drug effects also cannot be rule out. For example, *in vitro* studies found that TMZ application can modulate K+ currents in glioma cells (Yeh et al., 2016), and reduced dendritic complexity and decreased PSD-95 expression in cultured hippocampal neurons (Lomeli et al., 2020). If these effects extend to neural circuits *in vivo*, then it is possible that some of the findings observed could be partially mediated by unaccounted effects of TMZ through neurogenesis-independent mechanisms, such as altered neuronal excitability, synaptic remodeling, or inflammation. Future studies will be needed to disentangle TMZ's neurogenesis-dependent and neurogenesis-independent effects. However, it is important to mention that we found no difference in the number of convulsive seizures experienced by the TMZ- and saline-treated kindled rats arguing that the effects of TMZ cannot be accounted for by differentially effects on kindling. That said, there is clinical evidence that TMZ can improve seizure control in patients with low-grade glioma independent of any change in antiepileptic drug treatment regimen (Sherman et al., 2011). Given that aberrant neurogenesis has been linked to increased hippocampal excitability and development of spontaneous seizures, it is possible that the anticonvulsant effects of TMZ seen in human patients may partly arise from its ability to affect seizure-induced neurogenesis and associated network remodeling. These observations underscore the translational relevance of our findings and support the idea that future research aimed at optimizing TMZ treatment protocols to more selectively target neurogenesis-dependent and/or neurogenesis-independent mechanisms could offer a novel approach to mitigate epilepsy-related cognitive dysfunction.

## 6 Implications

Our findings corroborate and extend other studies that have used other drug-based methods or more selective genetic strategies to suppress neurogenesis and mitigate cognitive deficits associated with epilepsy. For example, Jessberger et al. (2007) found that chronic treatment with the HDAC inhibitor valproate blocked aberrant seizure-induced neurogenesis and prevented memory deficits in kainic acid-treated rats. Similarly, Cho et al. (2015) elegantly showed that genetic ablation of new neurons beginning 4 weeks before pilocarpine treatment reduced the development of chronic recurrent seizures in these animals and normalized impairments in object location memory. These findings—and those from the present study—support the hypothesis that suppressing seizure-induced neurogenesis can affect the development of disease progression and emergence of cognitive impairment in chronic epilepsy. However, there has been conflicting results. For instance, Zhu et al. (2018) found that the cytotoxic methylazoxymethanol acetate was effective in suppressing aberrant neurogenesis when given both before and after the induction pilocarpine-induced seizures, but did not prevent the development of learning deficits. However, these animals still experienced spontaneous seizures which could account for the ongoing cognitive deficits. These findings suggest that targeting seizure-induced neurogenesis while being effective in alleviating certain aspects of cognitive dysfunction, may not be sufficient on its own to halt broader epileptogenic processes. One issue with chemoconvulsant models is that they produce widespread and extensive neuronal cell death and injury along with a myriad of other molecular and cellular changes that may not necessarily be involved in the development or progression of epilepsy. This makes it difficult to disentangle the direct effects of seizure activity on neurogenesis from other pathological sequalae such as neurodegeneration and spontaneous seizures that can also impact hippocampal function and behavior.

In this regard, we believe that the gradual and controlled evocation of repeated seizure activity that occurs with kindling offers an advantage to evaluate how seizure-induced neurogenesis contributes to circuit reorganization and cognitive dysfunction. For example, unlike commonly used models that induce prolonged seizures through either the application of pilocarpine, kainic acid or intensive electrical stimulation, the progressive nature of kindling enables study of processes linked to seizure-induced plasticity and epileptogenesis without the presence of extensive neuronal injury and damage or the requirement of inducing status epilepticus (Sutula and Kotloski, 2017). In addition, unless a large number of stimulations are given (>150 to 200 stimulations), kindling does not typically evoke spontaneous seizures (Pinel and Rovner, 1978). While this could be considered a drawback in that the animals in this study did not go on to fully develop unprovoked seizures during the process of kindling and thus would not be considered truly epileptic, our findings suggests that many of the features of aberrant neurogenesis, such as ectopic dentate granule cells and adult-born neurons with abnormal morphology, appear to develop early in the process of epileptogenesis and do not necessarily require that the animal fully exhibits spontaneous seizure for them to occur. Our data also indicate that even in the absence of extensive hippocampal injury, aberrant neurogenesis appears sufficient to disrupt network functions that are known to depend on intact neurogenesis, such as pattern separation.

## 7 Conclusion

The results of this study add to growing evidence supporting the interpretation that aberrant neurogenesis contributes to hippocampal circuit remodeling that underlie the development of cognitive dysfunction associated with epilepsy. We also show that targeting aberrant neurogenesis with the use of a currently approved drug, temozolomide, can mitigate memory deficits seen with chronic seizure activity. These findings suggest that pharmacological targeting of seizure-induced neurogenesis may offer a novel therapeutic avenue for managing and treating behavioral and cognitive impairments in chronic epilepsy.

## Data Availability

The raw data supporting the conclusions of this article will be made available by the authors, without undue reservation.

## Supplementary material

Supplementary Figure 1 Histology. **(A)** Bright-field image showing the location of an implanted electrode. **(B)** Schematic representation (black dots) of the location of the electrode tips in the left basolateral amygdala of the kindled (*n* = 21) and TMZ-treated kindled (*n* = 19) rats that received stimulation. Distances are measured from bregma. AP coordinates were between −2.80 and −3.30 mm from bregma according to Paxinos and Watson (1998) stereotaxic rat brain atlas. Scale Bar, 1,000 μm.
