# Supplementary figures and images for: Inhibiting aberrant seizure-induced neurogenesis by temozolomide improves cognitive impairments associated with long-term amygdala kindling
Source: Front Neurosci. 2025 Sep 3;19:1626118. doi: 10.3389/fnins.2025.1626118 (PMC12440974; doi:10.3389/fnins.2025.1626118)

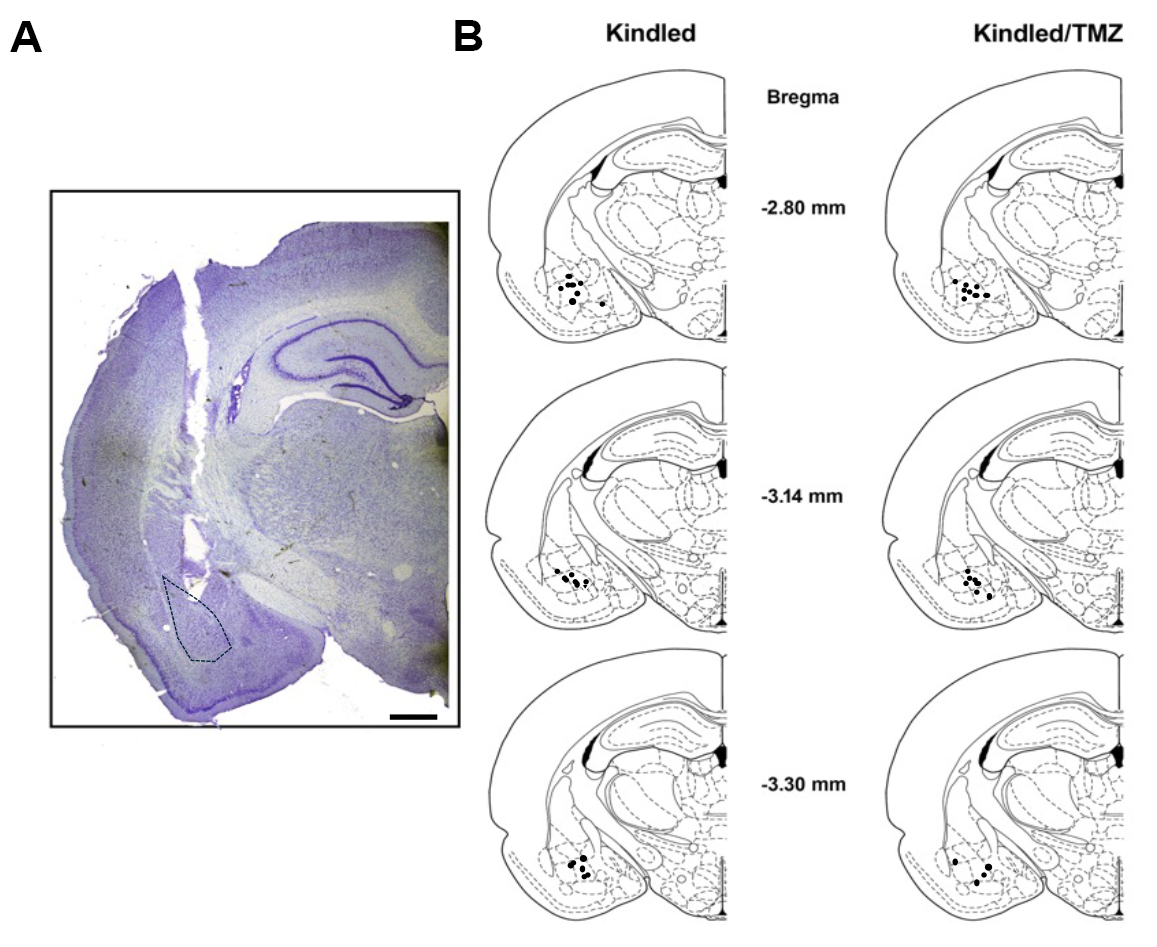

Supplement: Supplementary Figure 1 — Histology. (A) Bright-field image showing the location of an implanted electrode. (B) Schematic representation (black dots) of the location of the electrode tips in the left basolateral amygdala of the kindled (n = 21) and TMZ-treated kindled (n = 19) rats that received stimulation. Distances are measured from bregma. AP coordinates were between −2.80 and −3.30 mm from bregma according to Paxinos and Watson (1998) stereotaxic rat brain atlas. Scale Bar, 1,000 μm. [file Image_1.tif]
